# Discovery of rafoxanide as a novel agent for the treatment of non-small cell lung cancer

**DOI:** 10.1038/s41598-023-27403-y

**Published:** 2023-01-13

**Authors:** Ankang Hu, Jing Liu, Yonghui Wang, Maoyin Zhang, Yao Guo, Ying Qin, Tingya Liu, Yanjuan Men, Quangang Chen, Tingjun Liu

**Affiliations:** 1 Center of Animal Laboratory, Xuzhou Medical University, No. 209 Tongshan Road, Xuzhou, Jiangsu China; 2 Department of Respiratory Medicine, Xuzhou Central Hospital, No. 199 Jiefang South Road, Xuzhou, Jiangsu China; 3 School of Life Sciences, Xuzhou Medical University, No. 209 Tongshan Road, Xuzhou, Jiangsu China; 4 Department of Anesthesiology, The Affiliated Hospital of Xuzhou Medical University. No, 99 Huaihai West Road, Xuzhou, Jiangsu China; 5 Department of Neurology, The Affiliated Hospital of Xuzhou Medical University, No. 99 Huaihai West Road, Xuzhou, China; 6 Kangda College of Nanjing Medical University, No. 88 Chunhui Road, Lianyungang, China

**Keywords:** Cancer therapy, Oncology

## Abstract

Non-small cell lung cancer (NSCLC), which accounts for approximately 85% of all lung cancer cases, is associated with a poor outcome. Rafoxanide is an anthelmintic drug that inhibits tumor growth in certain malignancies. However, its impact on NSCLC remains unknown. In this study, we examined the effect of rafoxanide on NSCLC and dissected the underlying mechanism. The results showed that rafoxanide significantly inhibited the growth, invasion, and migration of NSCLC cells. Besides, rafoxanide can induce NSCLC cell apoptosis and cell cycle arrest in a dose-dependent manner. RNA-seq analysis revealed that genes associated with endoplasmic reticulum stress (ER) stress responses were activated. Mechanistically, we found Rafoxanide can induce ER stress and activate the unfolded protein response (UPR). Apoptosis was activated by excessive ER stress, and autophagy was activated to partially alleviate ER stress. In vivo, we found that rafoxanide inhibited the growth of A549 and H1299 xenograft mouse models without severe side effects. Collectively, the present study indicates that rafoxanide may be a candidate drug for the treatment of NSCLC.

## Introduction

Lung cancer is the most common cause of cancer-related death: more than 1.35 million deaths per annum in the word^1^. Approximately 85% of lung cancer cases are currently defined as non-small cell lung cancer (NSCLC). Surgical resection is generally considered as the most effective treatment for the patient with NSCLC, however, the treatment strategy depends on clinical staging, approximately 40% of patients were advanced stage at diagnosis, and lost the chance of surgery. Recently immune checkpoint inhibitors have become an important treatment for advanced lung cancer, however, the overall survival of NSCLC patients remains poor, with the 5-year survival less than 15%^2^.

The endoplasmic reticulum (ER) is a protein-folding checkpoint that provides a unique environment for the proper folding and maturation of newly synthesized proteins. Under certain stimuli, ER stress (ERs) activates the unfolded protein response (UPR) as an adaptive response for promoting the correct folding of proteins. ERs plays a critical role in the proliferation and viability of cancer cells.

Rafoxanide is an anthelmintic drug used for treating F. hepatica infection in sheep and cattle^3^. It is also used against gastrointestinal nematodes and nasal bot fly^4^. Andrea et al. showed the efficacy of rafoxanide as monotherapy and in combination with colistin against colistin-susceptible (Col-S) and Col-R *A.* baumannii, *P. aeruginosa* and *K. pneumoniae* strains^5^. Rafoxanide is used in combination with new antiviral drugs against adenovirus infections in immunosuppressed patients^6^. Recently, Rafoxanide has shown inhibitory effects on tumor growth. However, its impact on NSCLC remains to be examined.

In the present study, we found that rafoxanide can inhibit NSCLC cell growth and migration, and induce cell ERs. The UPR and autophagy were activated to eliminate the ERs, however, excessive ERs could induce cell apoptosis. Additionally, a xenograft mouse model was used to determine the effect of rafoxanide treatment on NSCLC, the results indicated that rafoxanide significantly suppressed the NSCLC tumor growth in vivo. Collectively, these findings indicated that rafoxanide might be a potential anti-cancer drug for NSCLC.

## Results

### Rafoxanide markedly decreases the growth and inhibits the invasion and migration of NSCLC cells

**Figure 1:**
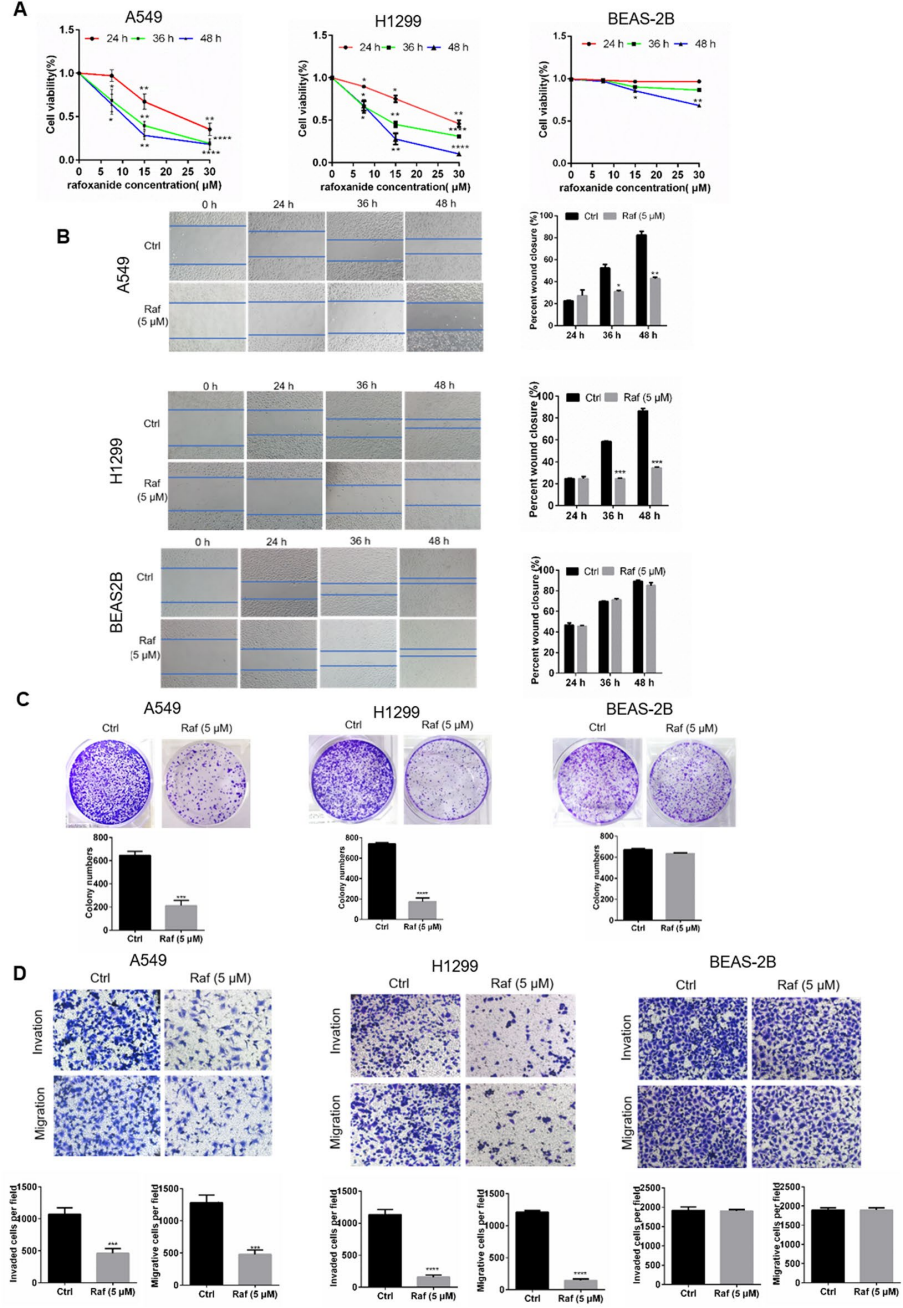
Rafoxanide significantly inhibits NSCLC cells proliferation, migration, and invasion. (**A**) A549, H1299, and HESA-2B cell were treated with rafoxanide at indicated concentration for 24, 36, and 48 h, and cell viability was assessed by the CCK8 assay. (**B**) Colony forming assay of different cells after exposure to rafoxanide (7.5 μM) for 14 days was analyzed. (**D**) The wound healing assay was performed to detect the effect of rafoxanide on A549, H1299, and HESA-2B cell migration at 5 μM. (**E**) The Transwell migration and Matrigel invasion assay were performed to assess cell migration and invasion after treatment with rafoxanide at 5 μM. Data are represented as mean ± SD of three independent experiments. *P < 0.05, **P < 0.01, ***P < 0.001, ****P < 0.0001.

To explore whether rafoxanide exerts its cytotoxic effects in NSCLC cells, we first sought to determine the effect of rafoxanide on A549 and H1299 cell viability through the CCK8 assay. We found that the viability of A549 and H1299 cells treated with rafoxanide was decreased significantly in a dose- and time-dependent manner, however, rafoxanide had little cytotoxicity in normal lung cells (BEAS-2B cell) (Fig. 1A). Besides, rafoxanide treatment also dramatically decreased the colony formation of NSCLC cells but not normal lung cells (Fig. 1B).

We next determined whether rafoxanide influences the migration of NSCLC cells. Cell migration rate was measured by wound healing assay, we found that rafoxanide significantly decreased the wound healing ability of NSCLC cells (Fig. 1C). Subsequently, we performed transwell migration and Matrigel invasion assays. In line with the wound healing assay, the invasion and migration capacity of NSCLC cells was obviously reduced (Fig. 1D). On the contrary, rafoxanide elicited little or no effect on cell motility of normal lung cells (Fig. 1C,D).

### Rafoxanide-induced inhibition of A549 cell growth is related to apoptosis and cell cycle arrest

**Figure 2:**
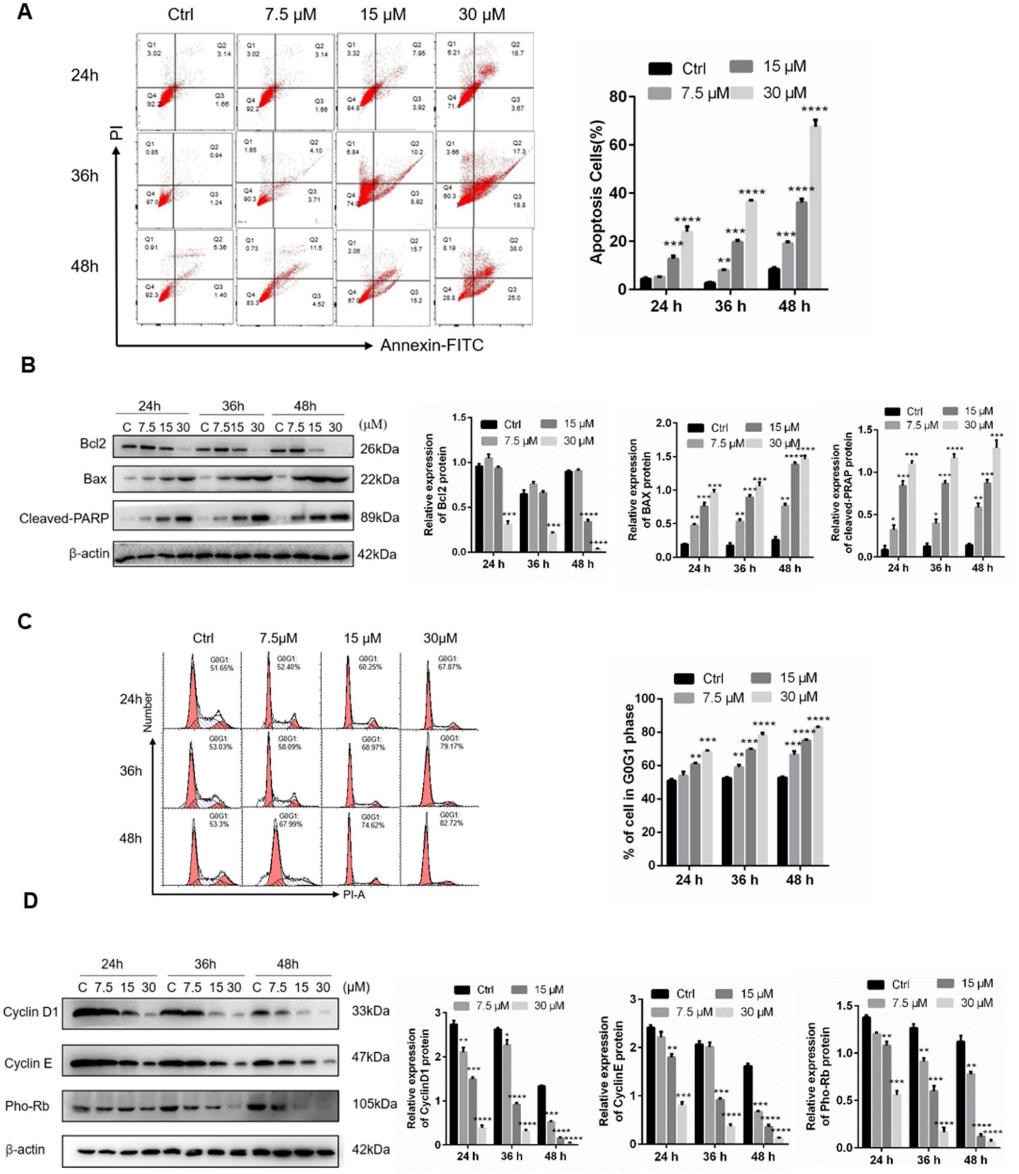
Rafoxanide promotes A549 cell apoptosis and induces cell cycle arrest. (**A**) A549 cells were treated with rafoxanide (0, 7.5, 15 and 30 μM) for 24, 36 and 48 h, and apoptosis was analyzed using flow cytometry. (**B**) Western blotting was performed to evaluate the protein expression levels of apoptosis-related proteins, including cleaved PARP, Bax, the anti-apoptotic protein Bcl-2, and phosphorylated Rb. The relative expression levels of cleaved PARP, Bax, Bcl-2, and phosphorylated Rb were analyzed with ImageJ based on the band intensities. (**C**) Flow cytometry was used to assess the effect of rafoxanide (0, 7.5, 15, and 30 μM) for 24, 36, and 48 h on the cell cycle. Columns show the percentage of the cell population in the G0/G1 phase. (**D**) Western blot analysis of the cell cycle-related proteins cyclin D1, cyclin E and phosphorylated Rb. The relative expression levels of cyclin D1, cyclin E and phosphorylated Rb were analyzed with ImageJ based on the band intensities. Data are expressed as the mean ± SD of three experiments. *P < 0.05, **P < 0.01, ***P < 0.001, ****P < 0.0001.

To elucidate how rafoxanide suppresses A549 cell viability, we determined whether rafoxanide could induce cell apoptosis and cell cycle arrest. A549 cells were treated with different concentration of rafoxanide for 24, 36, and 48 h and analyzed by flow cytometry. The results showed that rafoxanide induces apoptosis of A549 cells in a concentration- and time-dependent manner (Fig. 2A). Also, rafoxanide significantly promoted expression of the proapoptotic proteins, Bax and cleaved PARP, and reduced the expression of the anti-apoptotic protein Bcl-2 (Fig. 2B).

Using flow cytometric analysis, we assessed whether the cell growth inhibitory effects induced by rafoxanide are mediated through alterations in the cell cycle. A549 cells were treated with various concentrations of rafoxanide for different times. We observed that rafoxanide increased the proportion of A549 cells in G0/G1 phase, following treatment with increasing concentrations of rafoxanide, the cell number in the G0/G1 phase increased further (Fig. 2C). Meanwhile, the expression of cyclin D1, cyclin E and phosphorylated retinoblastoma (Rb) decreased significantly (Fig. 2D). These results indicate that rafoxanide inhibited A549 cell growth and induced cell apoptosis and cell cycle arrest.

### Rafoxanide induces endoplasmic reticulum stress in A549 cells

**Figure 3:**
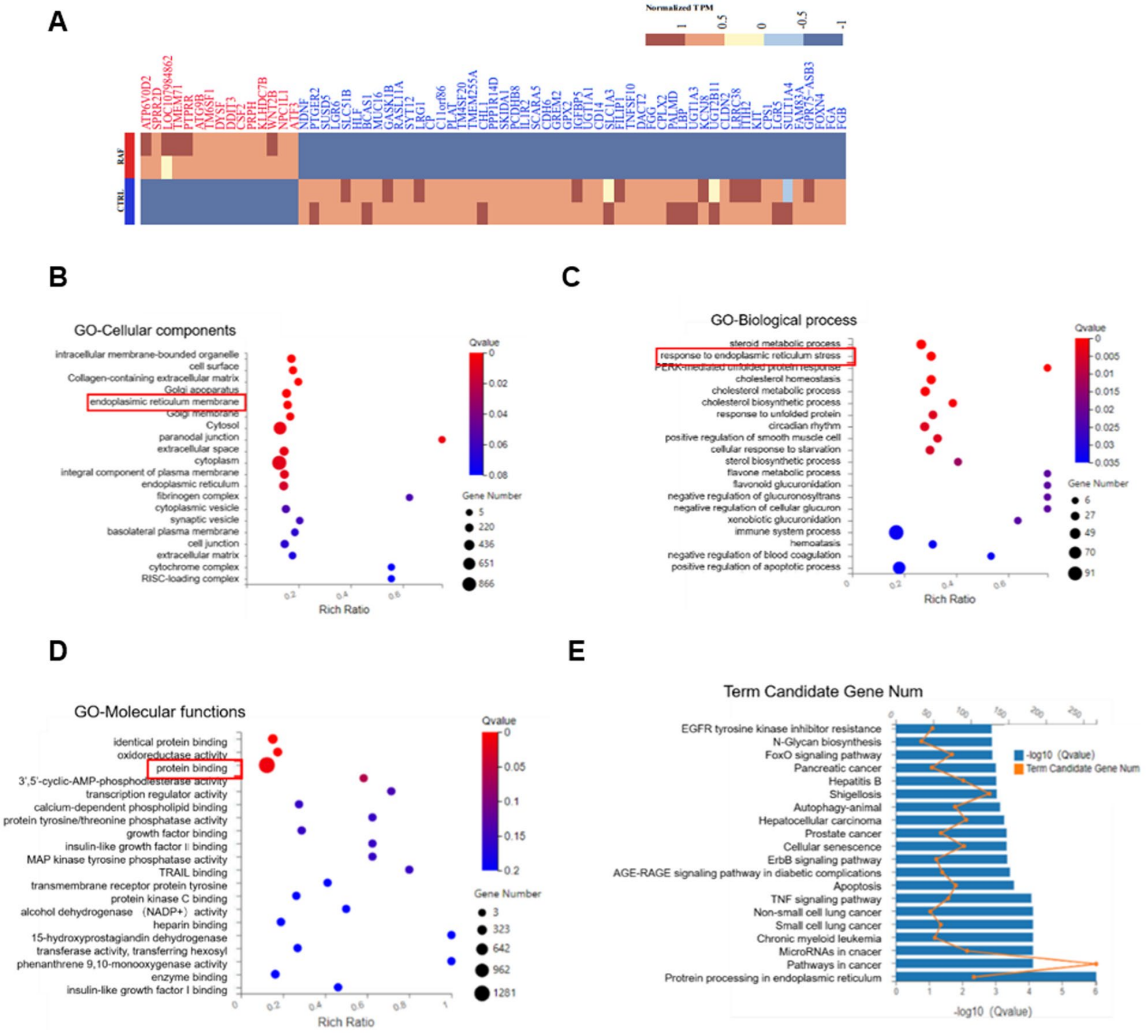
RNA-seq analysis of the ERs response upon rafoxanide treatment. (**A**) Heatmap of DEGs of A549 cells challenged with DMSO or rafoxanide. (**B–D**) GO classification of rafoxanide-regulated DEGs on the Dr. Tom platform (BGI-tech, China). The *x*-axis indicates the proportion and the y-axis indicates GO classification. (**E**) KEGG pathway enrichment analysis of DEGs. The *x*-axis indicates the proportion and the y-axis shows the KEGG pathway names.

To understand the anti-tumor mechanisms of the rafoxanide, RNA-seq was carried out using A549 cells after treatment with rafoxanide for 36 h. Gene expression profiles were compared. Differentially expressed genes (DEGs) between the rafoxanide and control groups under were shown in Fig. 3A. Gene Ontology (GO) and KEGG pathway enrichment analyses were subsequently conducted. GO analysis in the biological process category showed that the most significant differential terms were ‘steroid metabolic process’, ‘response to endoplasmic reticulum stress’, and ‘PERK-mediated unfolded protein response’. The most significant cellular components were ‘intracellular membrane-bounded organelle’, ‘cell surface’, ‘collagen-containing extracellular matrix’, ‘Golgi apparatus’, and ‘endoplasmic reticulum membrane’. The top molecular functions were ‘identical protein binding’, ‘oxidoreductase activity’, and ‘protein binding’ (Fig. 3B–D). KEGG pathway analysis demonstrated that the regulation of rafoxanide was associated with ‘Protein processing in endoplasmic reticulum’, ‘Pathways in cancer’, and ‘MicroRNAs in cancer’ (Fig. 3E). Taken together, these results strongly suggest that rafoxanide induces A549 cell apoptosis by affecting the function of the ER.

**Figure 4:**
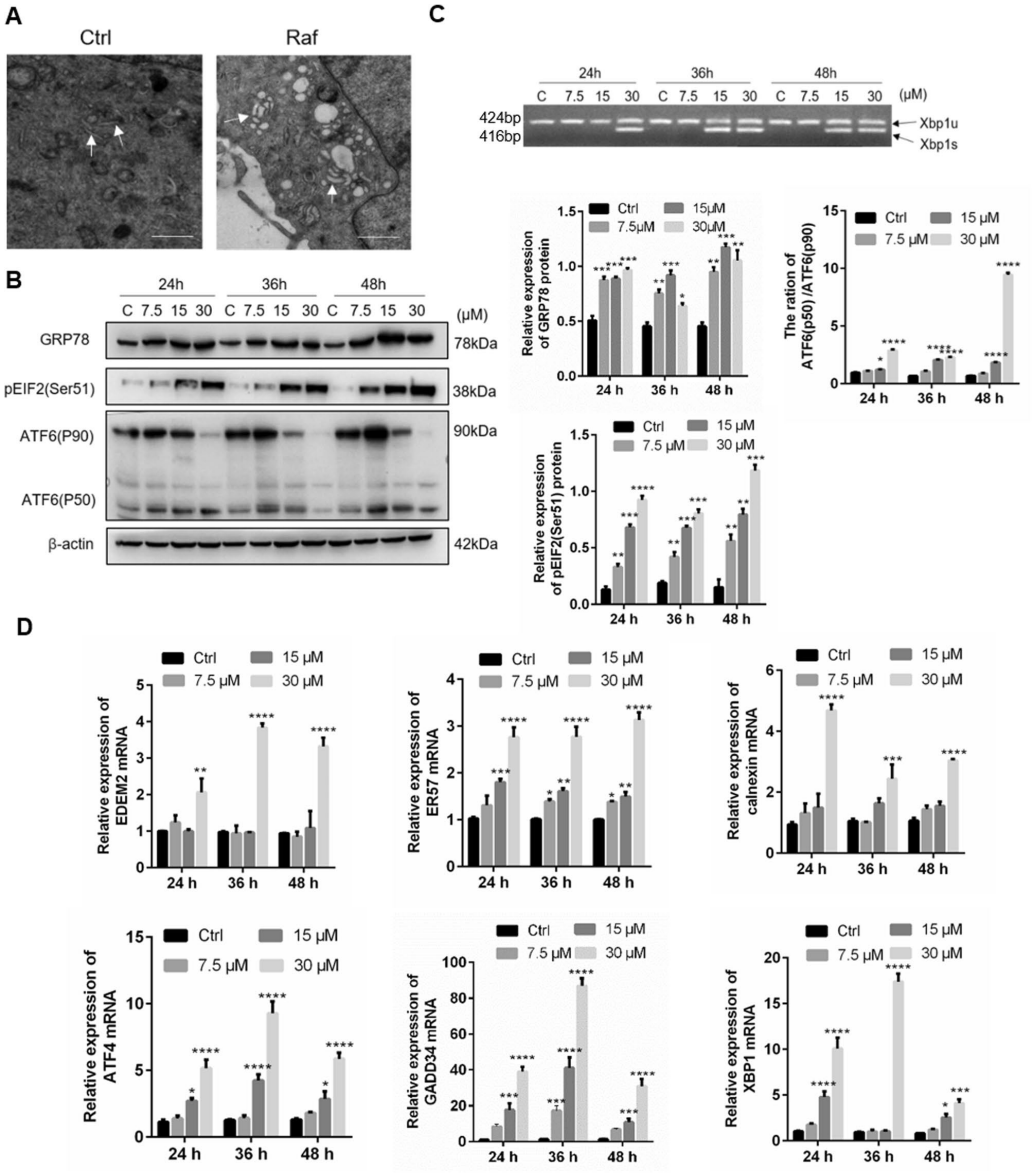
Rafoxanide promotes ERs and activates the UPR in A549 cells. (**A**) A549 cells were treated with 30 μM rafoxanide for 36 h. Cells were observed by electron microscopy. The ER (arrows) in cells is indicated. Scale bars, 500 nm. (**B**) A549 cells were treated with rafoxanide at different doses and cells were collected for western blot analysis at the indicated time points using antibodies against GRP78, pEIF2, and ATF6. β-actin was used as the loading control. The bar graph represents the quantitative analysis of western blots. (**C,D**) A549 cells were treated with rafoxanide (0, 7.5, 15, 30 μM) for 24, 36, 48 h. RNA was extracted from the treated and control cells. Both the unspliced and spliced forms of Xbp1 were analyzed by semi-quantitative RT-PCR. qRT-PCR was performed using primers specific for *ATF4*, *GADD34*, *XBP1*, *EDEM2*, *ER57*, and *calnexin*. *P < 0.05, **P < 0.01, ***P < 0.001, ****P < 0.0001.

ER swelling is a typical morphological change of cells during ERs. To evaluate whether ERs was induced after treatment with rafoxanide, cellular changes were observed by electron microscopy. We found that there was a significant expansion of the ER during rafoxanide treatment (Fig. 4A). Another hallmark of ER stress is the activated expression of ER chaperones. Here, we found that rafoxanide treatment upregulated the expression of GRP78 at various time points (Fig. 4B).

Three UPR signaling pathways, PERK, IRE1 and ATF6, participate in the elimination of ERs. During the UPR, PERK can phosphorylate eIF2α, the 90-kDa precursor of ATF6 is cleaved into a 50-kDa protein that functions as a transcription factor, and IRE1 splices a 26-bp intron from Xbp1u, resulting in an active Xbp1s. We found that after treatment with rafoxanide, the expression of phosphorylated eIF2α, cleaved ATF6, and Xbp1s was upregulated in A549 cells (Fig. 4B,C) and the downstream gene was activated (Fig. 4D). Collectively, we concluded that rafoxanide can induce ERs and active all three UPR pathways in A549 cells.

### ERs is related to rafoxanide-induced autophagy in NSCLC cells

**Figure 5:**
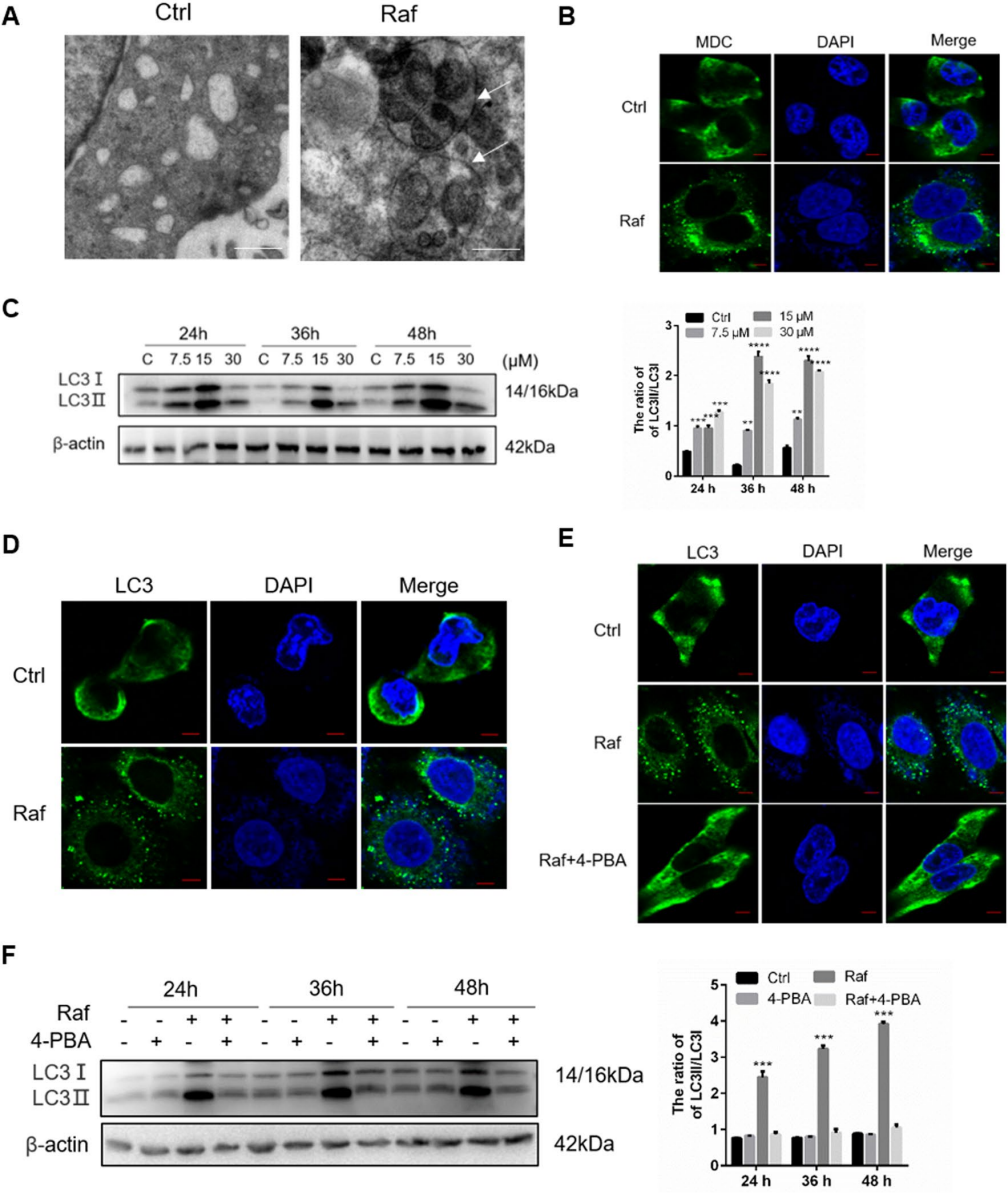
Rafoxanide-induced ERs contributes to autophagy in A549 cells. A549 cells were treatment with 30 μM rafoxanide for 36 h. (**A**) Cells were observed by transmission electron microscopy. Scale bars, 1 μm. (**B**) Cells were stained by MDC and observed by confocal microscopy. Scale bar, 10 μm. (**C,D**) A549 cells were treated with rafoxanide, cell lysates were obtained, and LC3 conversion was determined by western blotting. The LC3-II/LC3-I ratio was analyzed with ImageJ based on the band intensities. The formation of endogenous LC3 puncta was analyzed by fluorescence microscopy. Scale bar, 10 μm. (**E,F**) A549 cells were pretreated with 4-PBA for 2 h, and then cells were treated with rafoxanide. Formation of LC3 puncta was analyzed by immunofluorescence. Scale bar, 10 μm. Cell lysates were obtained and LC3 conversion was determined by western blotting. *P < 0.05, **P < 0.01, ***P < 0.001, ****P < 0.0001.

From the above results, we determined that rafoxanide can induce ERs, and the UPR was activated to alleviate ERs. Autophagy also generally regulated by UPR to attenuate the ERs. Previously studies revealed that rafoxanide promotes autophagy in gastric cancer cells^7^. To test whether rafoxanide-induced ERs further triggers autophagy in A549 cells, cellular changes was observed by electron microscopy. As shown in Fig. 5A, compared with control, rafoxanide treatment increased the number of autophagic vacuoles in A549 cells. The autophagic vacuoles were also stained by the monodansylcadaverine and observed by confocal microscopy. Similar to electron microscopy detection, more autophagic vacuoles were observed in rafoxanide treated cells (Fig. 5B). In addition, we detected the conversion of LC3-I to LC3-II and punctate accumulation of LC3. As shown in Fig. 5C,D, rafoxanide markedly promoted the conversion of LC3-I to LC3-II, and induced punctate accumulation of LC3.

To further determine whether rafoxanide-induced autophagy was triggered by ERs, we treated cells with 4-PBA (5 mM), an ERs inhibitor. As shown in Fig. 5E, treatment with 4-PBA decreased the punctate accumulation of LC3 induced by rafoxanide (Fig. 5E), and the LC3-II/LC3-I ratio was significantly ameliorated (Fig. 5F). These results suggest that ERs is responsible for rafoxanide-induced autophagy.

### Autophagy protects against rafoxanide-induced cell apoptosis in A549 cells

**Figure 6:**
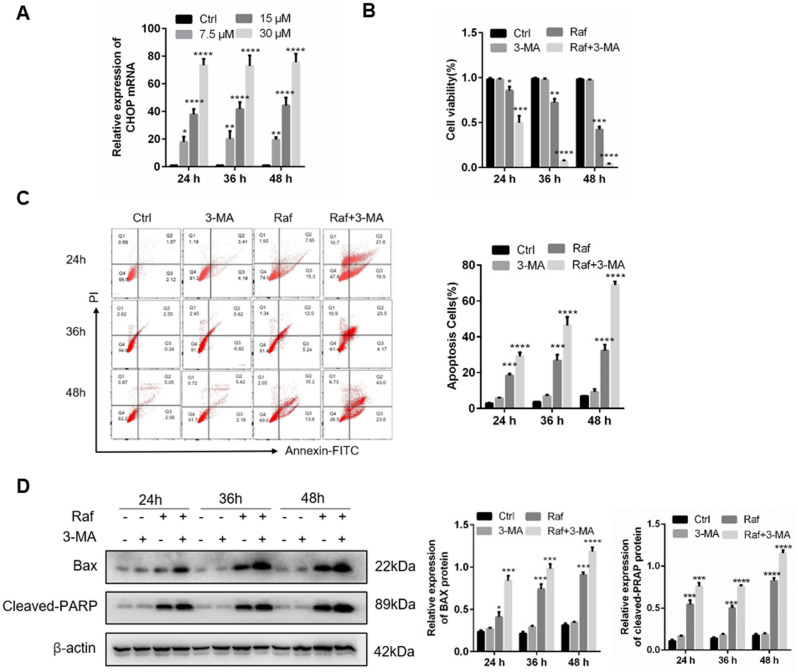
Autophagy induced by rafoxanide is involved in alleviation of rafoxanide-induced apoptosis. (**A**) A549 cells were treated with rafoxanide, and qRT-PCR was performed to detect mRNA expression of *CHOP*. (**B–D**) A549 cells treated with 30 μM rafoxanide for 24, 36, 48 h. 3-MA (5 mM) was added 2 h prior to rafoxanide. Cell viability was determined by the CCK8 assay. Apoptosis was analyzed by flow cytometry. Western blotting was performed to evaluate the protein expression levels of cleaved PARP and Bax. Data are expressed as mean ± SD of three experiments. *P < 0.05, **P < 0.01, ***P < 0.001, ****P < 0.0001.

We know that excessive ERs will lead to apoptosis. Elevated expression of CHOP is the hallmark of ERs-induced apoptosis. Hence, we detected the CHOP expression in rafoxanide treated A549 cells by qRT-PCR. As shown in Fig. 6A, CHOP expression was activated after treatment with rafoxanide. Autophagy plays a dual role in cell: anti-apoptosis or pro-apoptosis. To clarify the role of autophagy in rafoxanide-induced apoptosis, A549 cells were treated by 3-MA to inhibit autophagy, and then the cell viability was tested by CCK8 assays. The results showed that inhibition of autophagy further reduced the cell viability of rafoxanide-treated A549 cells (Fig. 6B). In addition, the combination of 3-MA and rafoxanide increased apoptosis of A549 cells (Fig. 6C). Consistent with the improvement of cell apoptosis, inhibition of autophagy by 3-MA significantly increased the expression of Bax and cleaved-PARP in A549 cells (Fig. 6D). All these results suggest that autophagy was activated to protects against ERs-induced apoptosis in rafoxanide-treated A549 cells.

### Rafoxanide suppresses tumor growth in xenograft mouse models

**Figure 7:**
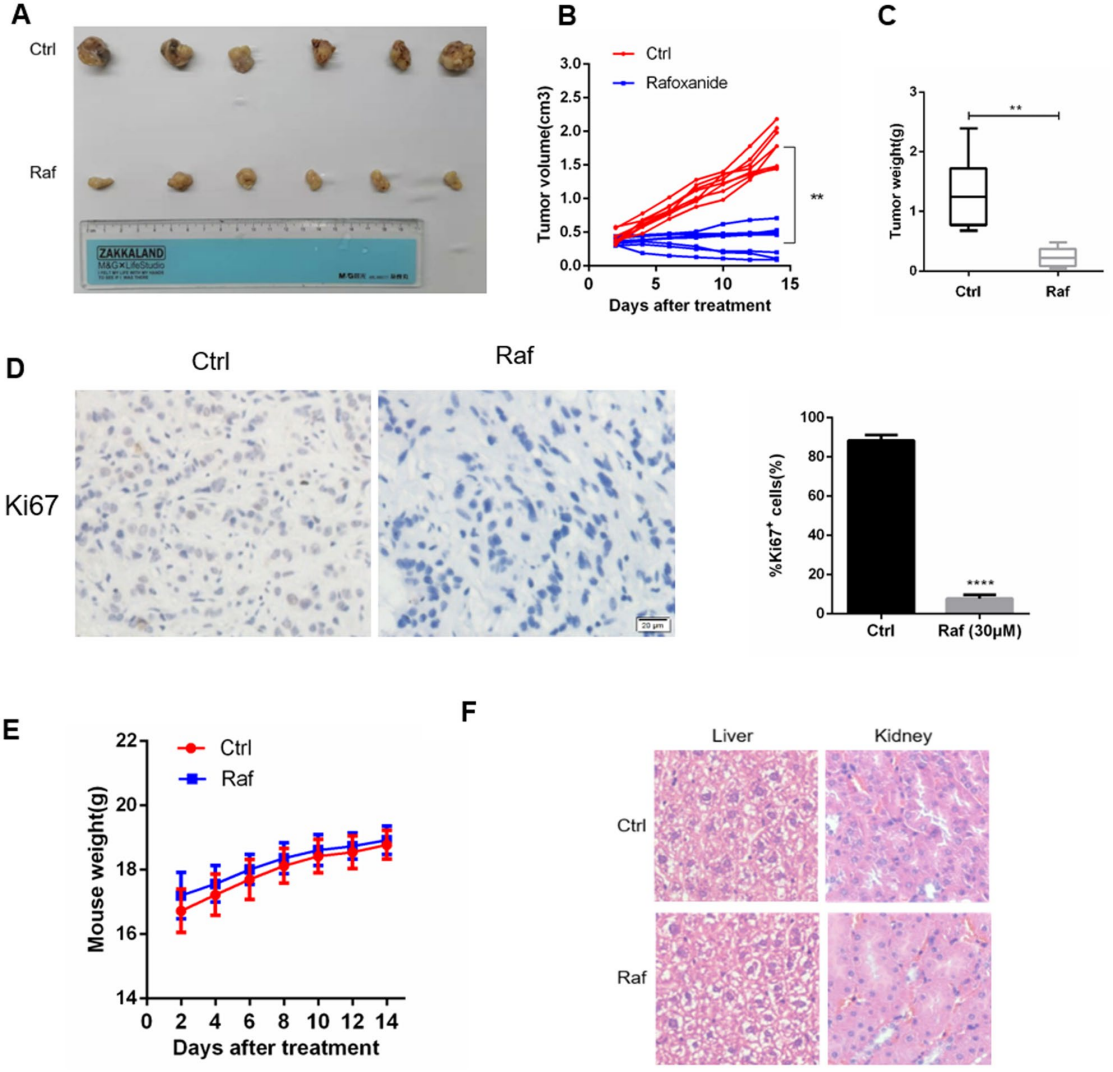
Rafoxanide exhibited anti-tumor activity in vivo. A549 cells were injected into BALB/c nude mice and when the tumor grew to 100 mm^3^, the mice were treated once a day with 15 mg/kg rafoxanide or vehicle**. (A–C**) Tumor volume was measured. 14 days later, the tumor samples were collected, photographed, and weighted. (**D**) Tumor tissues were stained with anti-Ki67. (**E**) Mice were weighted during therapy**.** (**F**) Histopathological analysis of major organs by H&E staining from the control and rafoxanide treatment groups. Data are expressed as mean ± SD of three experiments. *P < 0.05, **P < 0.01, ***P < 0.001, ****P < 0.0001.

To evaluate the therapeutic effect of rafoxanide in vivo, six-week old nude mice were subcutaneously injected with 5 × 10^6^ A549 cells to generate a xenograft mouse model. After 2 weeks, mice were treated with rafoxanide (15 mg/kg) or DMSO by intraperitoneal injection every other day for 14 days. The results clearly showed that the tumor volume growth rate of rafoxanide-treated mice was significantly lower than that of DMSO treatment (Fig. 7A,B). The tumor weight in the rafoxanide treated groups was also obviously reduced (Fig. 7C). Meanwhile, Ki67 immunohistochemical staining showed that the proliferation of tumor cells was significantly inhibited by rafoxanide (Fig. 7D). In addition, there were no significant body weight differences between the two groups (Fig. 7E) and there was no obvious pathological damage in the liver and kidney (Fig. 7F).

To further determine whether rafoxanide had any side effects, we analyzed liver and kidney function indicators in mouse serum in different groups using an autoanalyzer. The blood biochemical parameters are summarized in Table 1. Compared with Group A, no obvious changes were observed in the values of the ALT, ALB and AST in Groups B, C, and D. However, the kidney function indicators BUN and CREAT in Group D fluctuated slightly compared with Group A, although no statistically significant differences were observed. These results indicate that Rafoxanide might be a safe anti-tumor drug for NSCLC.

**Table 1:** Biochemical analysis at the end of rafoxanide treatment.

Parameters	Group A	Group B	Group C	Group D	Significance
AST (u/l)	85.4 ± 5.1	87.2 ± 4.3	86.3 ± 4.7	87.1 ± 3.5	^C^NS
ALT (u/l)	46.3 ± 4.2	45.4 ± 3.8	46.4 ± 5.1	45.8 ± 2.9	^C^NS
ALB (g/l)	24.1 ± 3.7	24.5 ± 2.9	24.5 ± 3.2	23.8 ± 4.4	^C^NS
BUN (mmol/l)	6.9 ± 1.3	7.2 ± 2.1	7.4 ± 1.1	7.5 ± 1.5	^C^NS
CRE (μmol/l)	23.7 ± 0.4	24.2 ± 0.9	24.5 ± 1.3	24.9 ± 1.7	^C^NS

Group A: No treatment.

Group B: Treated with 10 mg/kg rafoxanide.

Group C: Treated with 20 mg/kg rafoxanide.

Group D: Treated with 30 mg/kg rafoxanide.

Each value was expressed as mean ± SD (*n* = 6).

^*C*^*NS* statistically no significant difference at *P* < 0.05 by ANOVA-test. Statistically different from control.

## Discussion

The development and progression of lung cancer is a multi-step process. Certain tumors are caused by abnormal gene changes and protein expression, which subsequently lead to cell phenotypic transformation and progression^8–10^. Despite advances in targeted therapy and immunotherapy, the overall progression-free survival rate of lung cancer patients remains unsatisfactory. Rafoxanide is an antihelminthic drug that is used to combat fluke infections in ruminants^11^. Recent studies showed that Rafoxanide has inhibitory effects on tumors. For example, Shi et al. found that Rafoxanide is a potential candidate drug for human skin cancer by inhibiting CDK4/6^12^. Carmine and his colleague reported that Rafoxanide inhibits the proliferation of colon carcinogenic cells in vitro and in vivo^13^. Wanhe et al. demonstrated that diffuse large B-cell lymphoma cell apoptosis and cell cycle arrest are promoted by the anti-tumor activity of Rafoxanide^14^. In the present study, we assessed the effect of Rafoxanide on NSCLC.

Cell apoptosis and cell cycle arrest are major factors that inhibit tumorigenesis^15,16^. The results of flow cytometry showed that Rafoxanide significantly induced cell apoptosis and cell viability. The expression of pro-apoptotic proteins, cleaved PARP and Bax, was upregulated and the anti-apoptotic protein Bcl-2 was downregulated. In addition, Rafoxanide reduced the expression of cyclin D1，cyclin E and phosphorylated Rb, and promoted the accumulation of A549 cells in G0/G1 phase in a concentration-dependent manner. At equal concentration, compared with cell apoptosis rate (40%), the decline of cell viability is only 20%, hence, apoptosis only partly explains the reduction in cell viability.

To elucidate the mechanism of the effects of Rafoxanide on A549 cells, RNA sequencing was performed to identify genes, pathways, and functional terms regulated by Rafoxanide. The results indicated that Rafoxanide most probably activate ERs in A549 cells. The ER is the widest intracellular organelle that provides a unique environment for the synthesis, maturation, and proper folding of a wide range of proteins^17^. To alleviate ERs, the UPR is activated^18^. The UPR signaling pathway consists of three main branches involving the IRE1α, PERK, and ATF6α. Once activated by ERs, IRE1α phosphorylates itself and splices XBP1 mRNA to generate the splice variant XBP1s; PERK is autophosphorylated and activated. Active PERK leads to phosphorylation of the eIF2α, which attenuates protein translation, thereby reducing ER protein overload^19^; inactive ATF6 translocates to the Golgi apparatus, where it is cleaved by S1P and S2P into the active form. Active ATF6 binds to the ERSE in genes encoding ER chaperone proteins. All three branches could initiate the downstream signal transduction and activate the transcription of UPR target genes^20^. However, if the ERs was not eliminated, the UPR can induce apoptosis. Indeed, several studies revealed that ERs can be triggered under chemotherapeutic agents and lead to apoptosis. For example, in lung cancer cells treated with cisplatin, ERs will be induced, leading to apoptosis^21^. In this study, we also found that treatment with Rafoxanide significantly increased the expression of GRP78, and pronounced dilation of the ER lumen was observed. We concluded that ERs is activated in Rafoxanide treated cells. Different to cisplatin, which induced a low level of ERs^21^, even though the three branches of the UPR were activated to mitigate ERs, Rafoxanide induced seriously ERs, which can induce ERs-associated apoptosis.

Increasing evidence indicates that ERs promotes autophagy^22,23^. During autophagy, LC3-I interacts with phosphatidylethanolamine (PE) to form LC3-II, which facilitates the formation of autophagosomes^24^. Jia-Zhou Liu et al. reported that Rafoxanide induces autophagy in gastric cancer cells^7^. Consistently, we found that Rafoxanide treatment markedly elevated the number of LC3 puncta and increased the conversion of LC3-I to LC3-II. TEM revealed that Rafoxanide treatment markedly increased the number of autophagosomes compared with control cells. However, treatment with 4-PBA decreased the number of LC3 puncta and LC3-II expression. Taken together, these results indicate that ERs is related to Rafoxanide-induced autophagy in A549 cells. Autophagy is generally considered as a cell survival mechanism. However, recently autophagy has also been recognized as a cell death pathway^25^. In this study, we found that Rafoxanide-induced autophagy was involved in the elimination of ERs-induced apoptosis.

At last, in a xenograft mouse model, we found that intraperitoneal administration of Rafoxanide suppressed tumor growth significantly. These findings are consistent with previous data in a colon, gastric, and lymphoma tumorigenesis models^13,14^. In summary, the present findings suggest that Rafoxanide is a potential anti-cancer drug for the treatment of NSCLC.

## Materials and methods

### Cells and chemicals

The A549, H1299 and BEAS2-B cell line was purchased from the Cell Bank of Chinese Academy of Sciences (Shanghai, China) with short tandem repeat (STR) authentication in November 2017. Cells were cultured in DMEM (Meilunbio, China) supplemented with 10% heated-inactivated fetal bovine serum (FBS; GIBCO) and 1% penicillin/streptomycin (Beyotime, China) at 37 °C in a 5% CO_2_ incubator. Rafoxanide was purchased from Tokyo Chemical Industry. The ERs inhibitor 4-PBA and the autophagy inhibitor 3-MA were purchased from Med Chem Express (Monmouth Junction, NJ, USA).

#### Cell viability assay

Cells were seeded at a density of 1 × 10^5^ cells per well in 96-well plates and treated with various concentrations of Rafoxanide (7.5, 15 and 30 μM) for 24, 36 and 48 h. The control group was treated with DMSO. Cell viability was determined by a Cell Counting Kit-8 (CCK8) (Keygen Biotech, Nanjing, China).

### Apoptosis and cell cycle assay

A549 cells were seeded at a density of 1 × 10^6^ cells per well in 6-well plates and treated with various concentrations of Rafoxanide (7.5, 15 and 30 μM) for 24, 36 and 48 h. After washing the cells twice with PBS and centrifuging at 2000 rpm for 5 min, 2 × 10^6^ cells were washed in PBS and mixed with 5 μL Annexin V-FITC and 5 μL propidium iodide (PI) at room temperature in the dark for 5–15 min (Keygen Biotech, China). Apoptosis was analyzed using a FACSCalibur flow cytometer (BD, USA).

The cell cycle distribution was examined using the Cell Cycle Detection Kit (Nanjing KeyGen Biotech, China). A549 cells were seeded at a density of 1 × 10^6^ cells per well in 6-well plates and treated with various concentrations of Rafoxanide (7.5, 15 and 30 μM) for 24, 36, and 48 h. Cells were collected and washed in precooled PBS and fixed with precooled 70% ethanol at − 20 °C overnight. Then, 500 μL PI/RNase of a staining working solution prepared in advance was added in the dark at room temperature for 30–60 min. The cell cycle distribution was analyzed using a FACSCalibur flow cytometer (BD, USA).

### Immunofluorescence staining

A549 cells were seeded on cover glasses (NEST Biotechnology, Muxi, China) in 24-well plates, and treated with 30 μM Rafoxanide for 36 h. After washing with PBS, cells were fixed with 4% paraformaldehyde for 10 min and ruptured using 0.2% Triton X-100 for 10 min on ice. Then, cells were blocked with 0.5% BSA for 30 min and incubated with antibody against LC3 (Proteintech, USA) overnight at 4 °C, followed by incubation with Alexa Fluor 488-conjugated secondary antibody (Proteintech) for 1 h at 37 °C. After washing in PBS, cells were incubated with DAPI (Beyotime) for 10 min and fluorescence analysis was performed using a confocal laser scanning microscope and Zen2011 software.

### MDC staining

The Autophagy Staining Assay Kit with MDC was purchased from Beyotime. MDC is one of the most commonly used fluorescent probes in autophagy detection. A549 cells were seeded in 6-well plates and treated with 30 μM Rafoxanide for 36 h. The cells were washed with PBS and then stained with MDC for 30 min at room temperature. Then, 1 mL Assay Buffer was added and cells were washed twice. Fluorescence analysis was performed using a confocal laser scanning microscope and Zen2011 software.

#### Colony-forming assays

A single-cell suspension (5000 cells/well) was seeded in a 6-well plate and treated with 7.5, 15 and 30 μM Rafoxanide for 2 weeks. Cells were fixed with 1 mL 4% paraformaldehyde for 30–60 min and stained with 1 mL crystal violet staining solution for 20 min. Each colony was > 50 cells; the number of clones (0.3–1.0 mm in size) was counted to calculate the clone formation rate using ImageJ software. Nuclei were imaged under a STELLARIS laser scanning confocal microscope.

### Cell migration and invasion assays

Approximately 5 × 10^4^ cells in serum-free medium with various concentrations ofRafoxanide were placed into the upper chamber of an insert for migration assays (8-μm pore size, Millipore) and invasion assays with Matrigel (Sigma-Aldrich, USA). DMEM medium (600 µL) containing 10% FBS was added into the lower chamber. After 24 or 36, the cells that had migrated or invaded through the membrane were fixed with 4% paraformaldehyde and stained with crystal violet. The migrated or invaded cells were imaged and counted using ImageJ 1.53 (https://imagej.nih.gov/ij/download.html)^26^.

### Wound-healing assay

Approximately 1 × 10^5^ cells were seeded in DMEM culture medium containing 10% FBS. After 24 h, medium was removed, and cells were scratched with a pipette tip (time = 0 h), followed by incubation in serum-free DMEM culture medium with various concentrations of Rafoxanide at 37 °C and 5% CO_2_. Pictures were recorded under a microscope at different time points for quantification analysis.

### RNA extraction and RT-PCR analyses

Total RNA was extracted from the cells using TRIzol reagent (Tiangen) and reverse transcribed to cDNA. For RT-PCR, RNA was reverse transcribed to cDNA using a reverse transcription kit (Vazyme). Quantitative real-time PCR was performed in a Light-Cycler 480 (Roche) using SYBR Green I Master Mix (Roche). The sequences for primers are shown in Table 2.

**Table 2:** Primers used in this study.

Primer	Sequence 5' → 3'
ATF4-F	ATGACCGAAATGAGCTTCCTG
ATF4-R	GCTGGAGAACCCATGAGGT
GADD34-F	GGAGGAAGAGAATCAAGCCA
GADD34-R	TGGGGTCGGAGCCTGAAGAT
Xbp1-F	CCCTCCAGAACATCTCCCCAT
Xbp1-R	ACATGACTGGGTCCAAGTTGT
EDEM-F	CAAACATTCGAGTGGTAGGAGG
EDEM-R	CGCCATGAAGTAAGTTCACTGT
ER57-F	GCCTCCGACGTGCTAGAAC
ER57-R	GCGAAGAACTCGACGAGCAT
Clan-F	CCAAGGTTACTTACAAAGCTCCA
Clan-R	GGCCCGAGACATCAACACA
Clar-F	CCTGCCGTCTACTTCAAGGAG
Clar-R	GAACTTGCCGGAACTGAGAAC
CHOP-F	AGCTGGAACCTGAGGAGAGA
CHOP-R	TGGATCAGTCTGGAAAAGCA
GAPDH-F	TCATGACCACAGTCCATGCC
GAPDH-R	GGATGACCTTGCCCACAGCC

### RNA sequencing

A549 cells were seeded in 6-well plates and treated with Rafoxanide 30 μM for 36 h. A549 cells were collected and washed with PBS, incubated in TRIzol reagent, and subjected to BGI-tech. Genes meeting the selection criteria of |log_2_(fold change)|≥ 1 and Q < 0.001 were considered to be differentially expressed genes (DEGs). In addition, functional enrichment analysis, including GO and KEGG pathway enrichment analyses, were performed to identify which DEGs were significantly enriched at Bonferroni-corrected *P*-value ≤ 0.05 compared with the whole-transcriptome background. GO functional enrichment and KEGG pathway analyses were carried out by the Dr. Tom platform (BGI-tech, China).

### Transmission electron microscopy

A549 cells were seeded in 6-well plates and treated with 30 μM Rafoxanide for 36 h. Then, cells were collected and fixed in 3% precooled glutaraldehyde for 2 h. After washing 3 times in PBS, the cells were post-fixed in 1% osmium tetroxide for 1 h. Then, the cells were dehydrated in a graded series of acetone. Ultra-thin slices were generated and visualized by transmission electron microscopy (Tecnai G2 Spirit Twin).

### Western blot

Cells were collected, washed with cold PBS, and lysed with RIPA Lysis Buffer (Beyotime) on ice for 30 min. The protein concentration was determined using the BCA method (Beyotime). Equal amounts of protein were loaded onto a gel for separation and then transferred to a Hybond-NC membrane (VICMED). The membranes were blocked with 5% skim milk at room temperature for 1 h and cropped according to the molecular weight of the target protein. After that, the NC membrane was incubated with primary antibodies overnight at 4 °C. Primary antibodies were as follows: anti-Actin, anti-LC3, anti-GRP78, anti-Phospho-eIF2α, anti-ATF6, anti-BAX, and anti-Bcl2 were purchased from Proteintech. Anti-cleaved PARP, anti-cyclin D1, and anti-cyclin E were purchased from Abcam. Anti-phospho-retinoblastoma (Rb) was purchased from Cell signaling (#9308). The next day, membranes were washed and incubated with the appropriate horseradish peroxidase-conjugated secondary antibodies (1:3000, Proteintech) at room temperature for 1 h. The blots were detected using a chemiluminescence detection system (Bio-Rad, USA).

### Animal studies

All animal studies were performed under the Guide for the Care and Use of Laboratory Animals through the Laboratory Animal Ethics Committee of Xuzhou Medical University (No. 202112A502). All methods were carried out in accordance with relevant guidelines and regulations along with the ARRIVE guidelines (https://arriveguidelines.org/). Male BALB/c nude mice weighing 18–20 g and aged 5 weeks were purchased from Jiangsu Jicui Biotechnology Co., Ltd. A549 cells (5 × 10^6^) in 100 μL serum-free culture medium were injected into the right upper flank of nude mice. When the tumor volume reached 100 mm^3^, the 12 mice were randomly divided into the Rafoxanide and control groups (*n* = 6/group), and given either 15 mg/kg Rafoxanide or DMSO daily for 2 weeks. Tumor size and body weight were measured every other day. At the end of the treatment, the mice were sacrificed, and tumors were removed and weighed. All animal-related studies were approved by the Laboratory Animal Ethics Committee of Xuzhou Medical University (No. 202112A502) and carried out in compliance with the ARRIVE guidelines.

### Statistical analysis

Data are representative of three independent experiments performed in triplicate. Statistical analysis was performed by one-way ANOVA multiple comparison tests using SPSS version 22.0 software. *P* < 0.05 was considered statistically significant (*P < 0.05, **P < 0.01, ***P < 0.001, ****P < 0.0001) (Supplementary Figs. S1–S10).

## Supplementary Information

Supplementary Figures.

## Data Availability

All data generated or analyzed during this study are included in this published article and its supplementary information files.
