# Supplementary material for: Discovery of rafoxanide as a novel agent for the treatment of non-small cell lung cancer
Source: Sci Rep. 2023 Jan 13;13:693. doi: 10.1038/s41598-023-27403-y (PMC9839764; doi:10.1038/s41598-023-27403-y)
Supplement: Supplementary file 1 — Supplementary Figures. [file 41598_2023_27403_MOESM1_ESM.pdf]

Supplementary Materials

Figure S1 Original Western Blot images included in Figure 2B. Cropped WB bands displayed in Figure 2B are highlighted by a red box.

Figure S2 Original Western Blot images included in Figure 2D. Cropped WB bands displayed in Figure 2D are highlighted by a red box.

Figure S3 Original Western Blot images included in Figure 4B. Cropped WB bands displayed in Figure 4B are highlighted by a red box.

Figure S4 Original Western Blot images included in Figure 5C. Cropped WB bands displayed in Figure 5Care highlighted by a red box.

Figure S5 Original Western Blot images included in Figure 5F. Cropped WB bands displayed in Figure 5F are highlighted by a red box.

Figure S6 Original Western Blot images included in Figure 6D Cropped WB bands displayed in Figure 6D are highlighted by a red box.

Figure S7 Original electron microscopy images included in Figure 4A.

Figure S8 Original images of Figure 4C.

Figure S9 Original electron microscopy images included in Figure 5A.

Figure S10 Original immunofluorescence staining images and Monodansylcadaverine (MDC) staining included in Figure 5B, 5D, 5E.

Figure S1

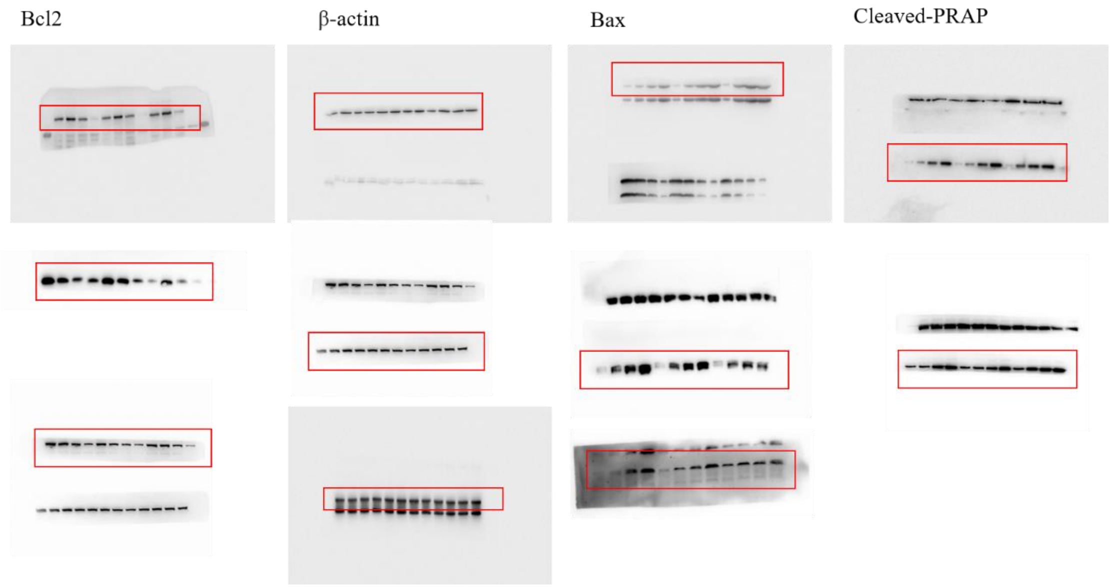

Figure S2

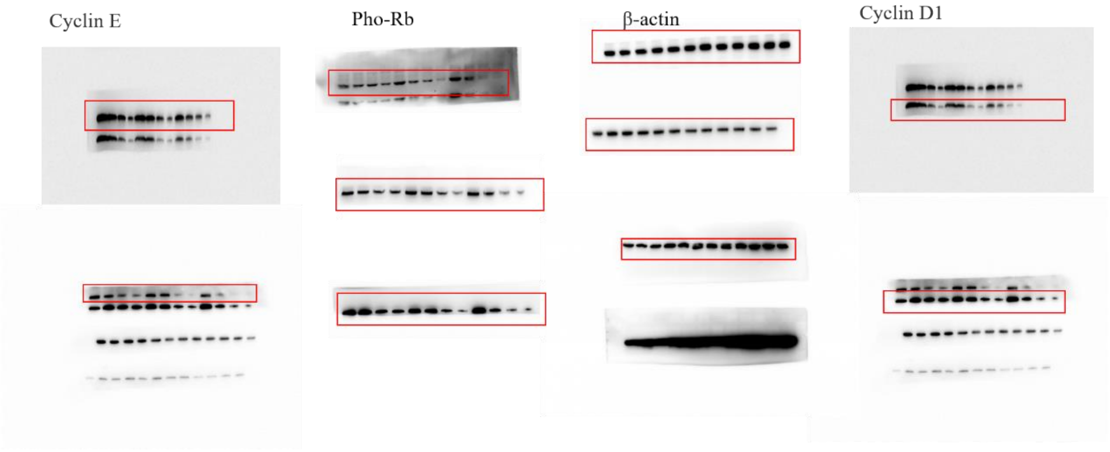

Figure S3

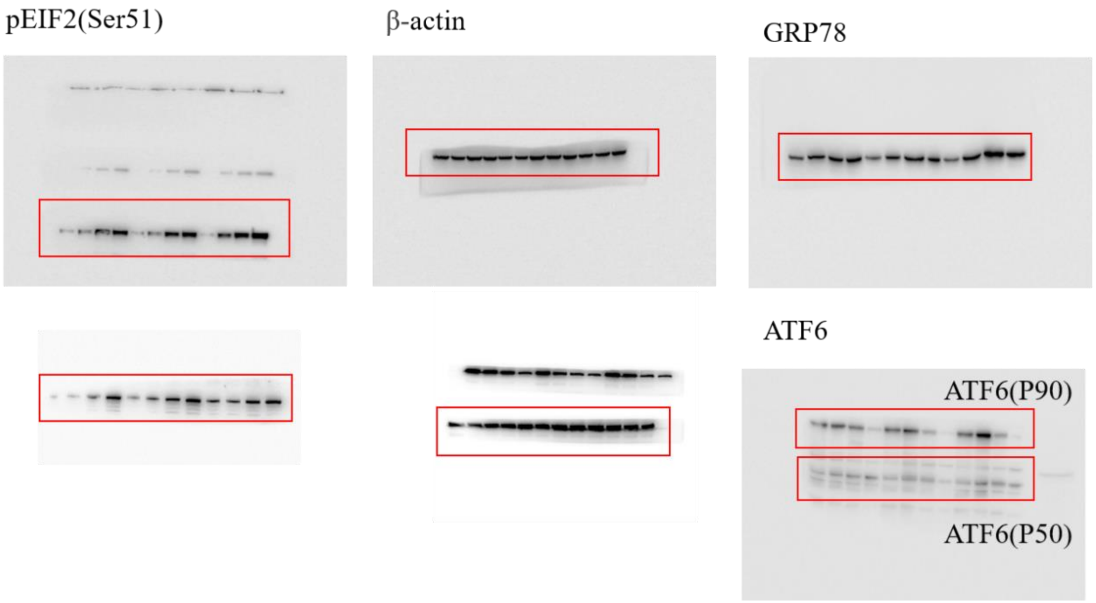

Figure S4

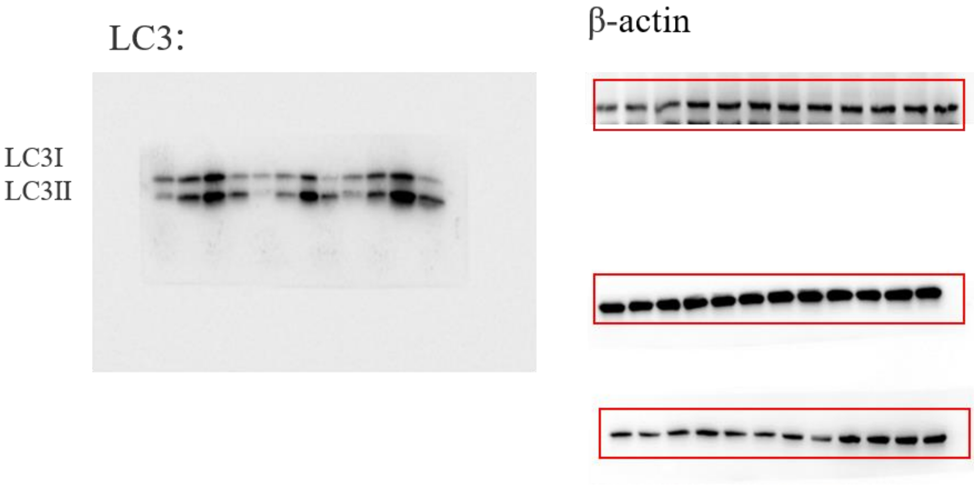

FigureS5

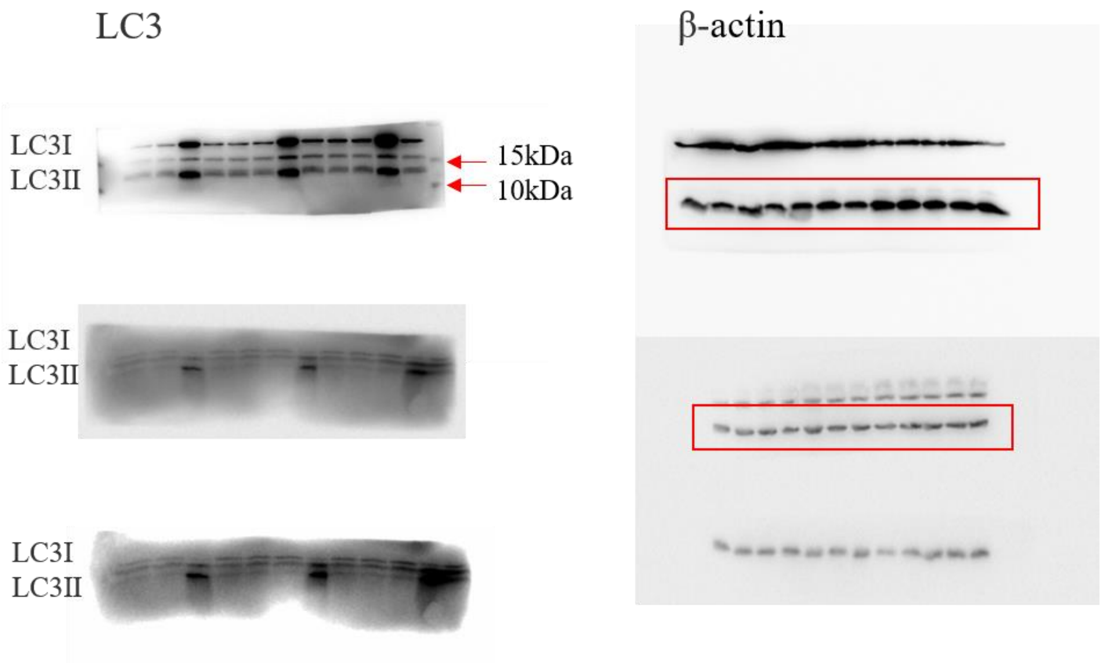

Figure S6

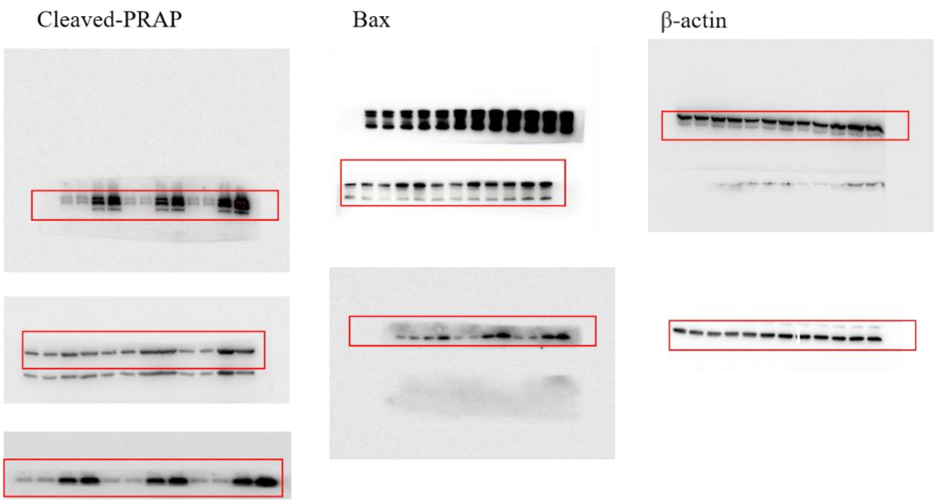

Figure S7

Ctrl

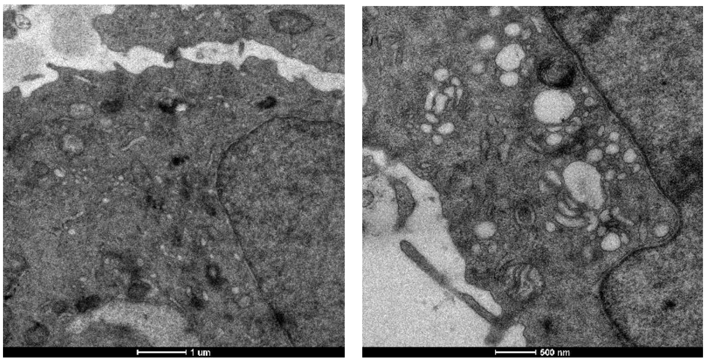

Rafoxanide

Figure S8

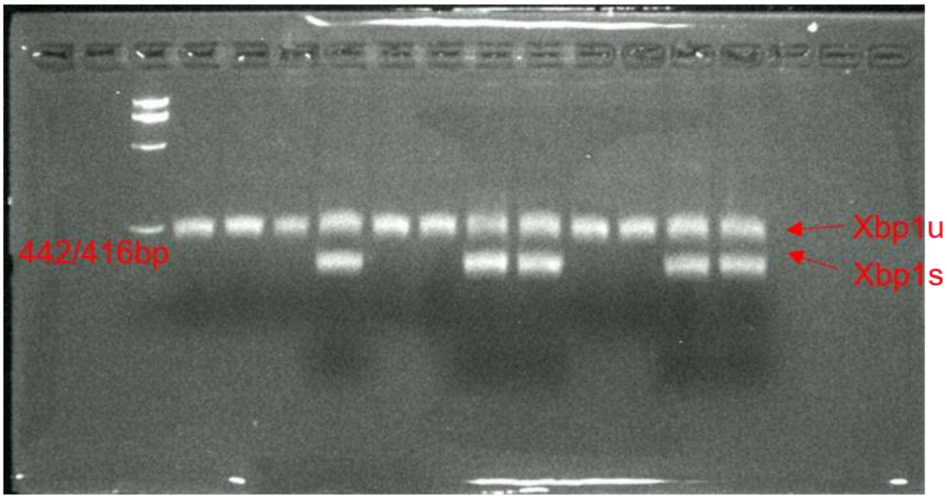

Figure S9

Ctrl

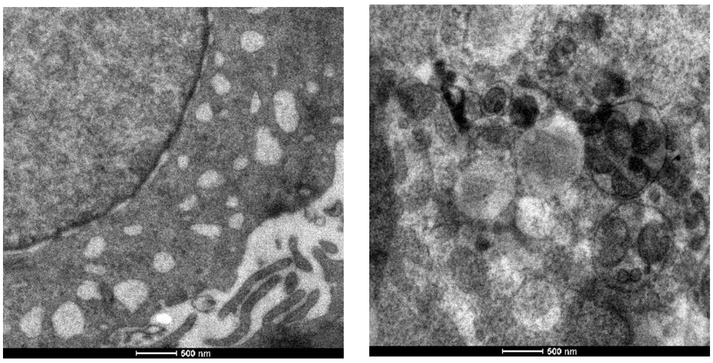

Rafoxanide

Figure S10

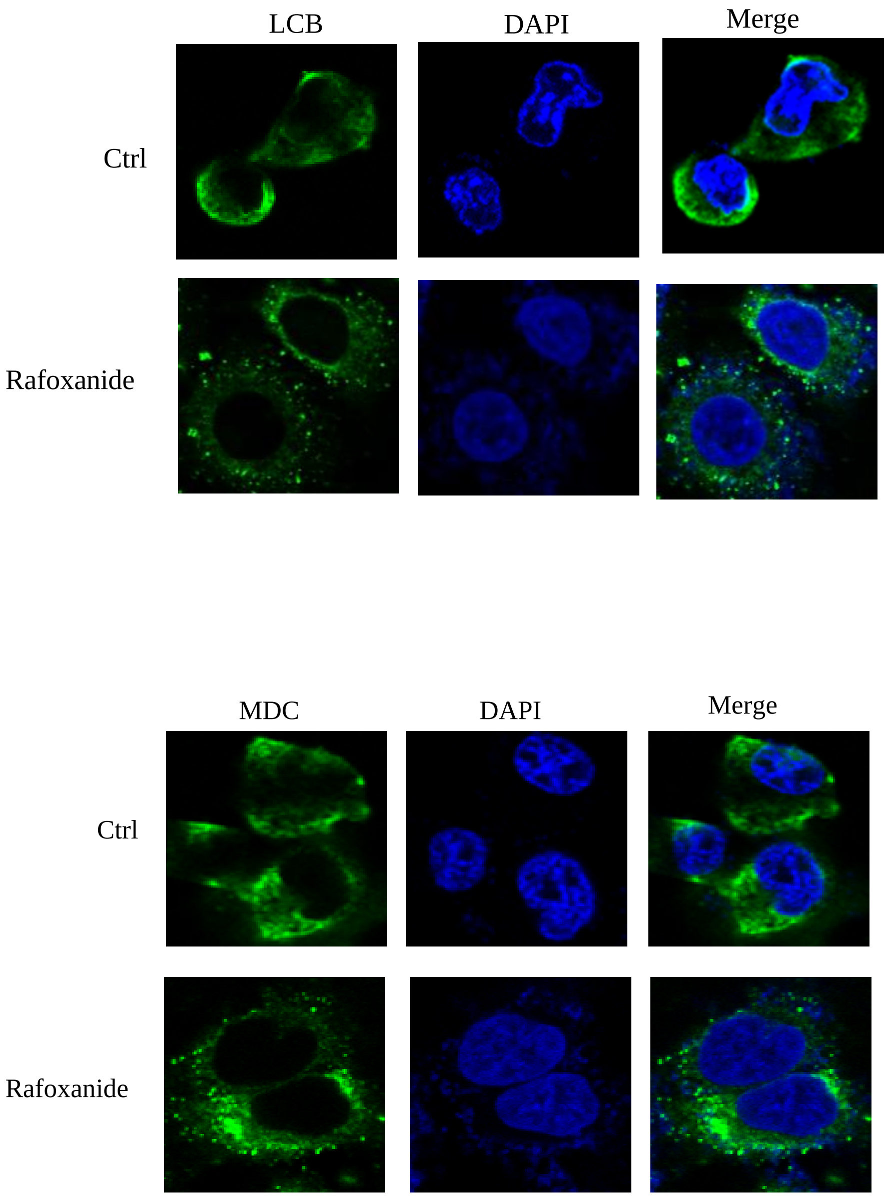
LCB
DAPI
Merge
Ctrl
Rafoxanide
MDC
DAPI
Merge
Ctrl
Rafoxanide

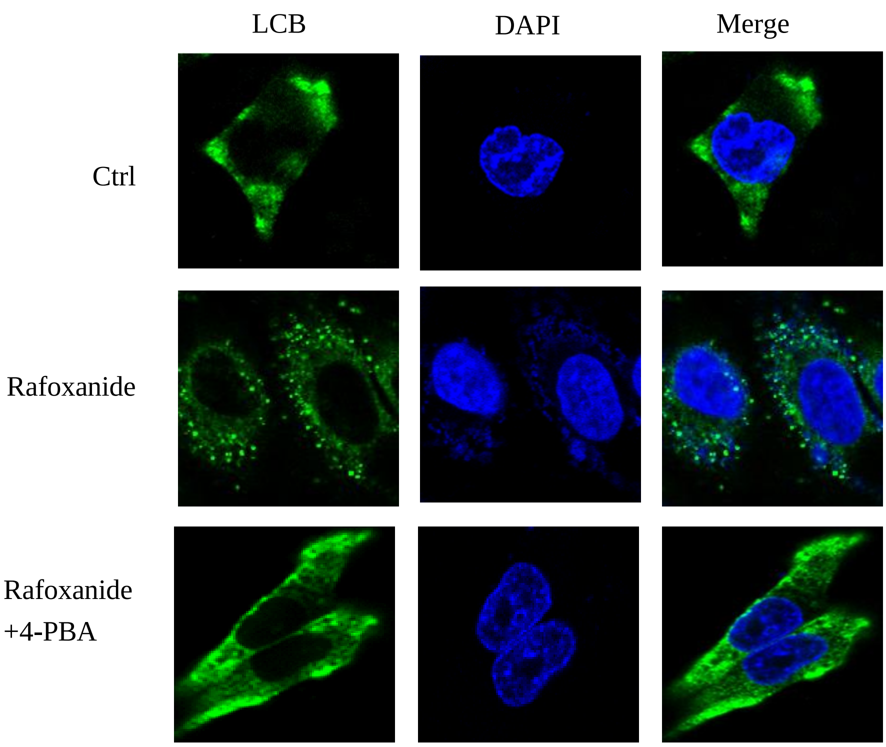
LCB
DAPI
Merge
Ctrl
Rafoxanide
Rafoxanide
+4-PBA
